# Human iPSC-Derived Dorsal Root Ganglion Organoid Modeling of Chemotherapy-Induced Peripheral Neuropathy

**DOI:** 10.3390/cells15080724

**Published:** 2026-04-19

**Authors:** Sybil C. L. Hrstka, Maya Jahnke, Kylie Meng-Lin, Sarah Lindorfer, Henry Noma, Ronald F. Hrstka, Nathan P. Staff

**Affiliations:** Department of Neurology, Mayo Clinic, Rochester, MN 55905, USA; hrstka.sybil@mayo.edu (S.C.L.H.); jahnke.maya@mayo.edu (M.J.); meng-lin.kylie@mayo.edu (K.M.-L.); s.lindorfer7@gmail.com (S.L.); h.h.noma@gmail.com (H.N.); hrstka.ronald@mayo.edu (R.F.H.)

**Keywords:** chemotherapy-induced neurotoxicity, iPSC, organoid, dorsal root ganglion

## Abstract

**Highlights:**

**What are the main findings?**

- Human iPSC-derived dorsal root ganglion organoids (iDRGOs) recapitulate key neuronal and glial features of native DRG tissue and support quantitative neurite outgrowth assays for modeling chemotherapy-induced peripheral neuropathy (CIPN).
- Bortezomib, paclitaxel, and vincristine induce dose-dependent neurite degeneration in iDRGOs, with distinct microtubule-associated protein (MAP2) responses compared to monolayer sensory neuron cultures.

**What are the implications of the main findings?**

- Three-dimensional organoid architecture and cellular heterogeneity influence measurable neurotoxic phenotypes, highlighting important differences between 2D and 3D human sensory neuron models.
- The iDRGO platform provides a physiologically relevant human system for mechanistic studies and therapeutic screening in chemotherapy-induced neurotoxicity.

**Abstract:**

Chemotherapy-induced peripheral neuropathy (CIPN) is a dose-limiting toxicity affecting 30–40% of patients treated with neurotoxic chemotherapy. Sensory symptoms arise from injury to dorsal root ganglion (DRG) neurons and their axons; yet, the underlying mechanisms remain incompletely understood. While human induced pluripotent stem cell (iPSC)-derived sensory neuron (iSN) monolayers have provided mechanistic insight, they lack the three-dimensional architecture and cellular heterogeneity of native DRG tissue. Here, we generated human iPSC-derived DRG organoids (iDRGOs) containing mixed neuronal and peripheral glial populations and established a quantitative neurite outgrowth assay to model chemotherapy-induced neurotoxicity in a 3D context. iDRGOs from three healthy donors were exposed to bortezomib, vincristine, or paclitaxel. All three drugs caused dose-dependent neurite outgrowth impairment without significant short-term changes in organoid size, consistent with early axonal injury. Vincristine reduced MAP2 levels when normalized to total protein, whereas bortezomib and paclitaxel showed divergent microtubule-associated responses compared to monolayer cultures. The developmental stage significantly influenced the baseline neurite outgrowth, highlighting the need for age standardization. These results establish iDRGOs as a physiologically relevant human platform that complements monolayer models for mechanistic studies and therapeutic screening in CIPN.

## 1. Introduction

Chemotherapy-induced peripheral neuropathy (CIPN) is a dose-limiting side effect of cancer treatment, affecting approximately 30–40% of patients exposed to neurotoxic chemotherapy. Clinically, CIPN most commonly manifests as a sensory neuropathy characterized by numbness, tingling, pain, and functional impairment, reflecting damage to dorsal root ganglion neurons (DRGs) and their distal axons [1]. Despite its prevalence and clinical impact, the mechanisms underlying CIPN remain incompletely understood. Multiple chemotherapeutic agents, including platinum compounds, vinca alkaloids, epothilones, taxanes, proteasome inhibitors, and immunomodulatory drugs, are known to cause CIPN, with platinum agents, bortezomib, taxanes, and vinca alkaloids being among the most commonly used in clinical practice. As cancer survivorship continues to improve, defining the mechanisms that drive chemotherapy-induced neural injury and developing therapies for CIPN will become increasingly important.

The biological effects of chemotherapy drugs on neurons have been studied in many different model systems, including human-derived, rat, and mouse DRG models, to better understand mechanistic causes that lead to CIPN [2,3,4,5,6,7]. These studies have demonstrated that multiple classes of chemotherapy drugs can directly injure peripheral sensory, motor, and autonomic neurons. Previously, we utilized human iPSC-derived sensory neuronal monolayer cultures (iSNs) as a human-based model to study molecular mechanisms driving sensory axonopathies [8,9]. While monolayer iSNs are a useful, and more physiologically relevant, model that mirrors similar neurotoxic effects observed within animal model systems [10,11,12], they are limited by their failure to recapitulate the three-dimensional spatial architecture and cellular diversity of native human DRGs [13]. This limitation merits the development of additional human iSN-derived models that can reproduce the complex architectural tissue of human DRGs [14].

Organoid systems have emerged as a system for modeling complex tissue architecture, as they capture dynamic interactions among multiple cell populations within a 3D environment [15,16]. While a fully mature in vitro model of the human DRG has yet to be established [6], recent studies have begun to address this gap by generating DRG organoids from iPSCs, enabling the investigation of different sensory neuronal subtypes [17]. Human iPSC DRG organoids have not, however, been utilized to study CIPN. The role of non-neuronal cells in CIPN is likely important, as the co-culturing of glial cells results in the altered maturation and differentiation of iSN [18]. Moreover, benefits have been observed in spot cultures of iPSC motor neurons, which are organoid-like, for studying the mechanisms underlying bortezomib-induced neurotoxicity [19]. Together, these studies highlight the potential of human organoid systems to elucidate the underpinnings of chemotherapy-induced neuronal injury within a three-dimensional framework.

In this study, we generated human DRG-organoids (iDRGO) to establish the effects of the neurotoxic chemotherapeutics of bortezomib, vincristine, and paclitaxel in a 3D culture system that contains diverse DRG cell types, and is thus likely more physiologically relevant. Furthermore, we compared the neurotoxic chemotherapeutic effects in the iDRGO model versus the monolayer iSN cultures.

## 2. Materials and Methods

### 2.1. Human iPSC Cell Culture, iSN Differentiation, and iDRGO Differentiation

iPSC lines were generated from the skin biopsies of three healthy adult donors from the Mayo Clinic Biotrust according to IRB-approved guidelines. Fibroblasts isolated from these samples were reprogrammed using transcription factors SOX2, OCT4, KLF4, and MYC via Sendai virus vectors (courtesy of CytoTuneTM-iPS 2.0 Sendai Invitrogen^TM^, Cat. No. A16518, Carlsbad, CA, USA). Karyotypically normal iPSC clones for each line that passed through quality control testing for pluripotency and germ layer differentiation were selected. Donor-derived lines used in this study included two healthy females (21-year-old; 6BT1 clone 1; 77-year-old; 1BT1 clone 139) and one healthy male (18-year-old; 100BT8 clones 214 and 217). iPSCs were cultured on LDEV-free Geltrex in mTeSR™1 medium (STEMCELL^TM^ Technologies, Cat. No. 85850, Vancouver, BC, Canada) at 37 °C with 5% CO_2_ and were passaged with ReLeSR™ (STEMCELL^TM^ Technologies, Cat. No. 100-0483, Vancouver, BC, Canada) at a 1:4 ratio every four days.

iPSCs were differentiated into dorsal root ganglion organoids (iDRGOs) following a previously reported approach with modification [17]. iPSCs were dissociated into single cells with Accutase and resuspended in mTeSR™1 supplemented with 5 µM Y-27632. Then, 9 × 10^3^ cells were seeded in low-adhesion V-bottom plates (Nunc^TM^ brand, Catalog No., 277143, ThermoFisher Scientific^TM^, Waltham, MA, USA) and centrifuged at 100× *g* for 5 min (days in vitro (DIV) -2). The following day (DIV -1), an equal amount of KSRb media (15% Knockout Serum Replacer (Gibco^TM^, Catalog No. 10828028, Grand Island, NY, USA), 1% MEM NEAA (Gibco^TM^, Catalog No. 11140-050, Grand Island, NY, USA), 1 mM Glutamax, and 100 µM β-mercaptenol in DME-F12 (Cytiva^TM^, Catalog No. SH30023.01, Marlborough, MA, USA) was added. During early patterning (DIV 0 and DIV 2), approximately half of the media was exchanged for KSRb media supplemented with 100 nM LDN193189 and 10 μM SB431542. In the early neuronal specification phase (DIV 4–DIV 8), half the media was exchanged every 48 h with KSRb|N2 media (Neurobasal media (Gibco^TM^, Catalog No. 21103-049, Grand Island, NY, USA), 1:100 N2 supplement (Gibco^TM^, Catalog No. 17502048, Grand Island, NY, USA), MEM NEAA, and 1 mM Glutamax) supplemented with 3 μM CHIR99021, 10 μM DAPT, and 3 μM SU5402 in addition to LDN and SB. During this timeframe, the proportion of N2 media gradually increased by 25% increments. On DIV 10, cultures were transitioned to neuronal maintenance medium supplemented with growth factors, NGF, BDNF, GDNF, and NT-3 (10 ng/mL each), and 200 µM L-ascorbic acid. Half of the medium was replaced every 2 to 3 days. iDRGOs between DIV 16 and DIV 37 were transferred to Geltrex-coated 24-well plates (Corning, Catalog No. 3530470, Corning, NY, USA) for downstream assays, with experiments initiated 24–48 h post plating. Assays with DRGOs > DIV 30 started approximately 48 h post plating.

iPSCs were differentiated into sensory neuronal monolayers (iSNs) as previously described [8,9]. Experiments were performed on iSNs ranging from DIV 25 to DIV 57 for neurite length assessments, and DIV 28 to DIV 51 for protein quantification (average age for both assays ~ DIV 41).

### 2.2. Chemotherapy Treatment

Bortezomib (LC Laboratories, Catalog No. B-1408, Woburn, MA, USA) and vincristine (Selleck Chemicals, Catalog No. S1241, Huston, TX, USA) were dissolved in DMSO (see Supplementary Table S2) to prepare stock solutions of 3.5 mg/mL and 5 mg/mL, respectively. Concentrations ranging from 6.25 nM to 100 nM were tested for both drugs, with the final DMSO concentration in the culture media maintained ≤0.002% which has been found to have no impact on neurite outgrowth in monolayer cultures of iSNs. Paclitaxel (Sigma-Aldrich, Catalog No. T7191, St. Louis, MO, USA) was reconstituted in a 50:50 (*v*/*v*) mixture of Kolliphor (MilliporeSigma, Catalog No. C5135, Burlington, MA, USA) and ethanol to generate a 5 mg/mL stock solution. The concentration range tested for paclitaxel was 62.5 nM to 1000 nM. Vehicle-control experiments with Kolliphor/EtOH ranged from 0.001% to 0.017%, which corresponds to the concentration range used for paclitaxel experiments. Untreated iDRGOs from each experimental batch served as the control condition. Nine experimental batches were included for bortezomib, nine experimental batches for vincristine, six experimental batches for paclitaxel, and five batches for kolliphor. Most experimental batches were completed with the 1BT1 and 6BT1 lines, with one experimental batch per condition representative for the 100BT8 line (clone 214). All experiments included four replicates per condition.

### 2.3. Immunofluorescence and Microscopy

iDRGOs were fixed with 4% paraformaldehyde for 1 h at room temperature and washed with D-PBS (Gibco^TM^, Catalog No. 14190144, Grand Island, NY, USA) three times before depositing in 30% sucrose (Sigma-Aldrich, Catalog No. S9378, St. Louis, MO, USA) solution at 4 °C overnight. iDRGO were embedded in 10% gelatin solution (Sigma-Aldrich, Catalog No. G2500, St. Louis, MO, USA), snap-frozen, and cryo-sectioned (12 µm thickness). Gelatin was removed from tissue sections by incubating warmed 0.1% tween-PBS (Sigma-Aldrich, Catalog No. P9416, St. Louis, MO, USA) solution on slides for 1 h. Heat-induced antigen retrieval was performed using citrate buffer (Sigma-Aldrich, Catalog No. S1804, St. Louis, MO, USA), and sections were blocked in 5% normal alpaca serum solution (Jackson ImmunoResearch, Catalog No. 028-000-121, Chester County, PA, USA) for 1 h. Primary antibodies were incubated overnight at 4 °C. Washing with D-PBS and secondary antibody incubation for 2 h followed the next day. Slides were mounted with VECTASHIELD^®^ PLUS Antifade Mounting Medium with DAPI (Vector Labs, Catalog No. H-2000-10, Newark, CA, USA). Antibodies are listed in Supplementary Table S2.

iSNs were fixed with 4% paraformaldehyde for 20 min at room temperature and permeabilized using 0.5% Triton X-100 for 15 min. Superblock was applied for 2 h, and primary antibodies were applied overnight at 4 °C. Washing with D-PBS and secondary antibody incubation for 2 h followed the next day, cells were counterstained with DAPI (BioLegend, Catalog No. 422801, San Diego, CA) and mounted with Prolong Gold Antifade Mountant (Invitrogen^TM^, Catalog No. P36962, Carlsbad, CA, USA). Antibodies are listed in Supplementary Table S2.

Phase images of iDRGOs and iSNs were acquired with an Incucyte SX5 (Sartorius Bioanalytical Instruments, Inc., Fremont, CA, USA). Images of iDRGOs were taken at 4X and images of iSNs were taken at 10X. All immunofluorescent images of iDRGOs were obtained using CKX41 confocal microscope equipped with a CP73 camera (Olympus, Hachioji, Tokyo). Images were taken with 10X/0.4W Olympus UPlanXApo objective, 20X/0.80W UPlanXApO, and 40X/1.15W Olympus UApO N340 water immersion objective. All immunofluorescent images of iSNs were obtained using Zeiss LSM 980 inverted laser scanning confocal (Carl Zeiss, Oberkochen, Germany). Images were taken with LD LCI Plan-Apochromat 40x/1.2 Imm Korr DIC M27 water immersion objectives. Images were processed using ZEN software (Carl Zeiss, Version ZEN Lite 3.12, Oberkochen, Germany).

### 2.4. Protein Quantification Analysis

Quantification of specific proteins of interest in cell lysates was determined using Simple Western immunoassay Wes system and chemiluminescent detection (ProteinSimple, San Jose, CA, USA), a capillary-based immunoassay platform in which separation and immunodetection occur within capillaries. Data are generated as electropherograms and instrument-rendered virtual lane images using Compass software (version 6.3.0, ProteinSimple, San Jose, CA, USA). Four iDRGOs were combined for each sample. iDRGOs were harvested from 24-well plates using RIPA Lysis buffer (ProteinSimple, Catalog No. 040-483, San Jose, CA, USA) with 1X protease and phosphatase inhibitors (ThermoFisher Scientific^TM^, Cat. Nos. 87786 and 78446, Waltham, MA, USA). Lysates were centrifuged at 1000× *g* for 5 min, supernatant was collected, and 3 ul of lysate was loaded into each capillary. Assays were then processed and quantified using Compass for SW (version 6.3.0), with protein abundance calculated from integrated peak areas. Due to cell population heterogeneity in iDRGOs, GAPDH served as a reference for protein normalization in iDRGO samples. Monolayer cultures of iSNs were >85% in purity, allowing for the use of Tuj-1 for the normalization of protein levels. Samples treated with chemotherapy drugs were normalized to untreated samples to assess the effects of drug treatment on levels of Tuj-1, PGP9.5, and MAP2. Antibodies and additional reagents used for the protein assays are listed in Supplementary Table S2. The original, uncropped, and unadjusted Wes-rendered images are provided in Supplemental Materials.

### 2.5. Quantitative RT-PCR

RNA was extracted from iDRGOs using TRIzol (ThermoFisher Scientific^TM^, Catalog No., Waltham, MA, USA), chloroform (Sigma-Aldrich, Catalog No., St. Louis, MO, USA), and isopropanol precipitation. cDNA was synthesized from RNA using iScript gDNA cDNA Synthesis Kit (BioRAD, Catalog No. 172503, Hercules, CA, USA). Quantitative RT-PCR was performed using iTaq Universal SYBR Green One-Step Kit (BioRAD, Catalog No. 1725150, Hercules, CA, USA). Primers used are in Supplementary Table S1. Each sample represents 1 biological replicate.

### 2.6. Neurite Length Analysis

Time-course images acquired with an Incucyte SX5 (Sartorius BioAnalytical Instruments, Inc., Fremont, CA, USA) were exported and analyzed in CellProfiler [20] (version 4.2.8). A standardized 15-module analysis pipeline was created for systematic image processing, object identification, neurite detection, and quantitative measurements. iDRGO area coverage and neurite area coverage was quantified for single images and exported for downstream analysis. Accurate neurite detection required identification of the primary object, the iDRGO. For images in which cell migration out of the iDRGOs prevented successful automatic iDRGO identification, iDRGOs were manually enhanced by outlining the iDRGO using Paint (version 11.2302.20.0, 2023, Microsoft Corporation, Redmond, WA, USA) prior to CellProfiler analysis. The CellProfiler pipeline created for this project is available upon request.

iDRGO size and neurite area coverage measurements were used to determine the fold change from the start of the assay (0 h) and at 12 h intervals up to 96 h. A single endpoint measurement (48 h) was selected for experiments with chemotherapy drugs. One replicate for each condition per experimental batch was analyzed. Area measurements were normalized to the control condition within each batch to account for inter-batch variability.

Neurite length measurements for monolayer iSNs were based on neurite degeneration after 72 h of exposure to chemotherapy drugs. Phase images were analyzed using the Incucyte NeuroTrack software package (Incucyte controller version 2024A). All three lines were represented in the assays, and two clones of the 100BT8 line were used. Each experimental batch assessed neurite length measurements in treated and untreated iSNs with four replicates per condition and four images per replicate. Neurite length measurements were normalized to t = 0 h for each image set, and the 72 h time point was used for a comparison among all conditions. One experiment per clone was completed for all substances tested.

### 2.7. Statistical Analysis

Statistical analyses of iDRGO and iSN experiments were performed using R (version 4.4.2) (https://www.R-project.org, accessed 1 November 2025 to 15 February 2026). A Kruskal–Wallis test followed by Dunn’s post hoc test with Holm correction was used to assess pairwise comparisons for changes in iDRGO size, neurite area coverage, and protein level measurements. A linear mixed-effect model was used to determine significant changes in iDRGO area and neurite area measurements for a time course series based on four iDRGOs. A Dunn’s post hoc test with Bonferroni correction was used to assess neurite length measurements for monolayer iSNs. The box plots in the figures illustrate the median and both upper and lower quartiles, with whiskers extending to 1.5 times the interquartile range. Statistical significance was defined as a *p*-value or adjusted *p*-value of *p* < 0.05.

## 3. Results

### 3.1. Generation and Characterization of Dorsal Root Ganglion Organoids (iDRGOs)

**Figure 1:**
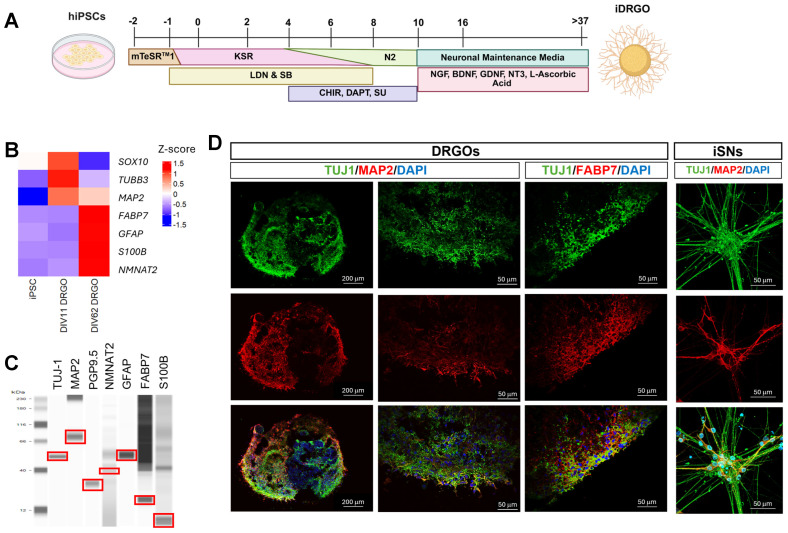
Characterization of iPSC-derived dorsal root ganglion organoids (iDRGOs). (**A**) Schematic of iDRGO differentiation. iPSCs were seeded in mTeSR™1 media and transitioned to KSR media with LDN and SB small molecules from DIV 1–4. From DIV 4–10, KSR media is added in decreasing percentage, N2 media was added in increasing percentage, and CHIR, DAPT, and SU small molecules were added. At DIV 10, media was changed to neuronal maintenance media with growth factors (NGF, BDNF, GDNF, NT3) and l-ascorbic acid. (**B**) Gene expression of *TUBB3*, *SOX10*, *FABP7*, *GFAP*, *S100*, and *MAP2* for iPSCs, DIV11 iDRGOs, and DIV 62 iDRGOs. (**C**) Representative Wes-rendered protein quantification of DIV > 40 iDRGO neuronal (Tuj-1, MAP2, PGP9.5, and NMNAT2) and glial cell (GFAP, FABP7, and S100B) markers. Representative protein quantification assay data shown is compiled from multiple independent assays. (**D**) Representative immunofluorescent images of DIV 86 iDRGOs and DIV 24 iSNs. iDRGOs stained for Tuj-1, MAP2, and FABP7 and sensory neurons (iSNs) stained for neuronal markers Tuj-1 and MAP2.

The protocol by Mazzara and colleagues established and characterized dorsal root ganglia (DRG) organoids that resemble a transcriptomic signature of native, human DRGs [17]. Following this protocol, three iPSC lines were differentiated in aggregate form as outlined in Figure 1A. At DIV 10, iDRGOs were maintained in neuronal media supplemented with growth factors in non-adherent V-bottom plates. iDRGO diameters ranged from 700 mm to 1 mm, and the size was independent from iPSC seeding counts of 2.3 × 10^3^–9 × 10^3^. All assays were based on iPSC seed counts of 9 × 10^3^.

iDRGOs were characterized for cell population heterogeneity and maturity using qPCR, protein assays, and immunofluorescent staining for markers representative of neuronal and non-neuronal cell populations. The qPCR expression of neuronal and peripheral glia lineage markers in iPSCs, DIV 11, and DIV 62 iDRGOs indicate that a neuronal population is established early in the differentiation process and that the satellite glial and Schwann cell populations require additional time for lineage commitment. The differentiation of iPSCs to DIV 11 iDRGOs led to an increase in the relative expression of the neural-progenitor-associated transcription factor *SOX10* which subsequently declined in DIV 62 DRGOs, indicating the presence of immature neural- and glial-progenitor-like cell populations at the early stages of iDRGO differentiation. Given our prior work dissecting the role of microtubule dynamic dysfunction in bortezomib-induced neurodegeneration in iSN, *MAP2* and *NMNAT2* were included as markers for neuronal maturity and axonal integrity [9]. *TUBB3* and *MAP2* expression were enriched in DIV 11 iDRGOs, whereas *NMNAT2* expression gradually increased over the course of differentiation with the highest expression levels in DIV 62 iDRGOs. DIV 62 iDRGOs also had a higher relative expression of satellite glial markers *FABP7, GFAP, and S100B* relative to the earlier stages of differentiation (Figure 1B). Thus, these transcript expression results indicate that iDRGOs’ cellular populations progressively transition from neural crest progenitor cell types toward a mix of mature sensory neurons, satellite glia, and Schwann cell types, characteristic of native DRG development [8]. Protein level expressions of the same neuronal and glial markers were also assessed. Neuronal markers Tuj-1, MAP2, PGP9.5, and NMNAT2, and satellite glial cell markers FABP7, GFAP, and S100B were detected in DIV 40 iDRGOs (Figure 1C). The presence of these markers in DIV 40 iDRGOs indicates that these organoids contain diverse populations of both neuronal (MAP2+, NMNAT2+, TUBB3+, and PGP9.5+) and satellite glia and Schwann cells (S100B+, FABP7+, and GFAP+) populations.

The differentiation of iPSCs in aggregate form often results in a heterogeneous distribution of cell populations within an organoid model. Immunofluorescence staining was performed on DIV 80 iDRGOs to assess the localization of the neuronal and non-neuronal cell populations. Both types of cell populations were intermixed within the iDRGOs. In general, the neuronal cell populations tended to be located outside of the core region of iDRGOs as shown by the localization of βIII-tubulin (Tuj-1) and MAP2 (Figure 1D), as well as PGP9.5 (Supplementary Figure S1). The satellite glial and Schwann cell populations were uniformly distributed throughout the iDRGOs as indicated by FABP7 and GFAP staining (Figure 1D and Supplementary Figure S1). Of note, signs of necrosis were absent, and the nuclear morphology appeared normal in DIV 80 iDRGOs, which indicates adequate nutrient availability to support cell viability. Altogether, these results demonstrate that DIV 40+ iDRGOs’ cellular composition contains sensory neurons and peripheral glial cell types that are characteristic of native DRGs.

### 3.2. Developmental Stage Influences Neurite Outgrowth and iDRGO Size

**Figure 2:**
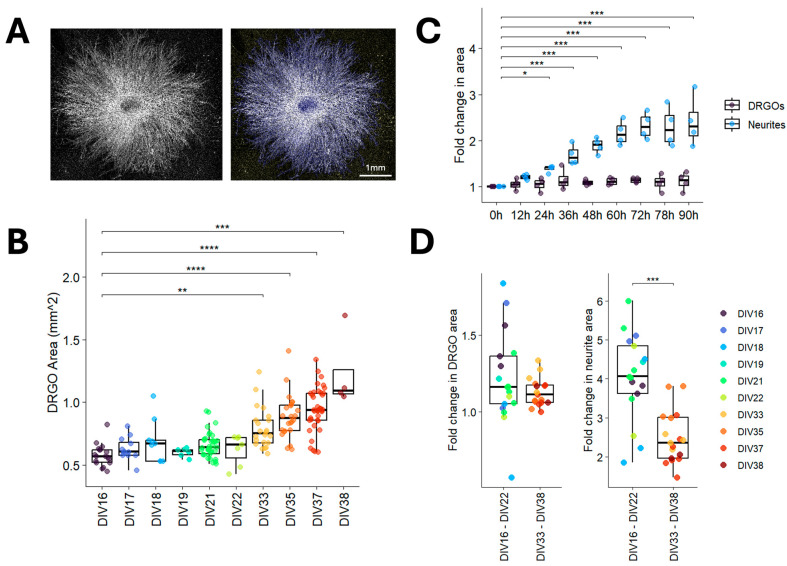
Characterization of iDRGO size and neurite outgrowth. (**A**) Representative image of neurite outgrowth after 48 h from a DIV 16 iDRGO (left) analyzed with CellProfiler (right). Object overlays are depicted in blue. Scale bar is 1 mm. (**B**) iDRGO sizes determined by area coverage from phase images for DIV 16 to DIV 38. Significant increases in area are observed between DIV 16–22 and DIV 33–38. Significance was determined by a Dunn’s test with Holm correction. ** *p*-adj < 0.005; *** *p*-adj < 0.0005; **** *p*-adj < 0.00005. (**C**) Comparison of iDRGO area and neurite outgrowth over a 90 h assay in DIV 37 iDRGOs. Cell cluster area measurements were unchanged. Pairwise comparisons among the different time points indicate area coverage of neurites was significant after 24 h (linear mixed-effects model; *n* = 4). * Pr(>|t|) < 0.02; *** Pr(>|t|) < 0.00003. (**D**) DIV 16–22 iDRGOs exhibit a significantly higher rate of neurite outgrowth compared to DIV 33–38 iDRGOs over a duration of 48 h. Significance was determined using Student’s *t*-test. *** *p* < 0.0005.

The next objective was to assess the iDRGO cellular growth dynamics and neurite establishment for different ages of iDRGOs ranging from DIV 16 to DIV 38. iDRGOs were seeded into individual plate wells to enable a radial array of neurite outgrowth and then analyzed using a CellProfiler pipeline to measure the area coverage of iDRGO cell aggregates and neurites (Figure 2A). Because our methodology did not incorporate mitotic inhibiting agents to reduce the proliferation of non-neuronal cells, we analyzed the iDRGO area coverage across various developmental stages. Untreated iDRGOs from all experimental batches in this study were included in the analysis. DIV 16–22 iDRGOs were similar in size but were significantly smaller than DIV 33–38 iDRGOs, suggesting the proliferation of the non-neuronal cell populations in the iDRGOs within this timeframe, which is after the neurons are post-mitotic (Figure 2B).

An assessment of iDRGO size and neurite outgrowth over a 90 h timeframe was completed with DIV 37 iDRGOs (Figure 2C; *n* = 4). The iDRGO area coverage remained stable throughout the assay with no significant differences detected across time points. In contrast, significant neurite outgrowth was observed commencing 24 h after iDRGO plating. Although the interpretation of these results is limited by the relatively small sample size, the observed dynamics suggests that neurite outgrowth occurs independently of soma size.

The probable cellular composition changes in DIV 16–22 iDRGOs compared to DIV 33–38 iDRGOs raises the issue of whether the developmental stage affects neurite outgrowth assessments in a 48 h assay. Using the untreated iDRGO measurements from all experiments, the fold change in iDRGO size and neurite outgrowth was normalized and compared for these different groups (Figure 2D). iDRGO size was steady regardless of age. However, neurite outgrowth was less robust in DIV 33–38 iDRGOs, indicating that the expansion and maturation of non-neuronal cells may influence the behavior of the neuronal cell population.

### 3.3. Chemotherapy Drug Exposure Affects DRGO Neurite Outgrowth

The chemotherapy-induced inhibition of neurite outgrowth has been well-documented in embryonic and adult DRG rodent models [7,21,22]. However, neurite degeneration in response to chemotherapy has not yet been studied in human iDRGO models.

To investigate neurotoxic effects on iDRGO size and neurite outgrowth, iDRGOs were exposed to bortezomib, paclitaxel, and vincristine across varying doses. Prior experiments have found that a 0.1% concentration of DMSO has no effect on neurite outgrowth (Supplementary Figure S2). As such, bortezomib and vincristine were dissolved in DMSO, with the highest DMSO concentration being ≤0.002% in the cell culture medium. Untreated iDRGOs and Kolliphor-treated iDRGOs, the vehicle used to solubilize paclitaxel, served as controls.

**Figure 3:**
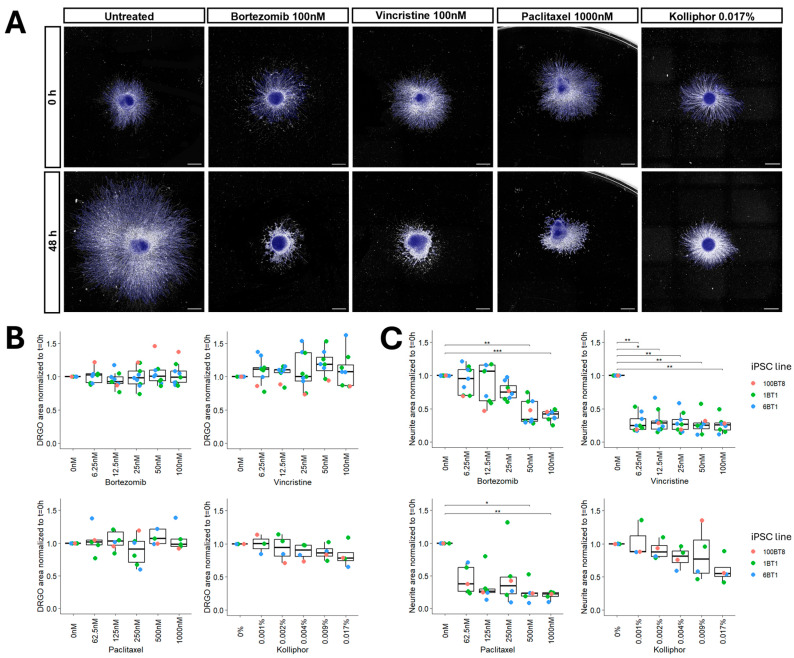
Neurite outgrowth assays following chemotherapy drug exposure. (**A**) iDRGOs treated with chemotherapy drugs at 0 h and 48 h time points. Scale bars = 200 µm. (**B**) iDRGO size comparisons following chemotherapy exposure. The fold change for each iDRGO area measurement was determined for a 48 h treatment and was normalized to untreated iDRGOs for each experimental batch. No significant size changes were observed in all trials. Sample numbers: bortezomib (*n* = 11), vincristine (*n* = 9), paclitaxel (*n* = 6), and kolliphor (*n* = 5). (**C**) Neurite outgrowth impairment following chemotherapy drug exposure. Fold change in neurite area was determined for a 48 h treatment for each iDRGO and normalized to untreated iDRGOs for each experimental batch. Significance was determined using a Dunn’s test with Holm correction. * *p*-adj < 0.05; ** *p*-adj < 0.005; *** *p*-adj < 0.0005.

The fold change in iDRGO and neurite area coverage in response to chemotherapy drug exposure was determined by comparing the 48 h image to the initial image acquired at the start of the assay for each iDRGO. All measurements were normalized to the average measurements obtained from untreated iDRGOs, eliminating the need to consider iDRGO age for neurite outgrowth assays. With this framework, exposure to the highest drug concentrations exhibited a marked reduction in neurite area coverage by 48 h (Figure 3A). Furthermore, representative images also illustrate that CellProfiler was able to accurately distinguish and segment boundaries of true iDRGO soma and neurite area.

Exposure of iDRGOs to chemotherapy drugs or Kolliphor had no effect on the overall size of the iDRGO cellular aggregates during the 48 h timeframe, suggesting that the gross iDRGO structure and size was largely preserved (Figure 3B). In contrast, neurite outgrowth was significantly affected in a drug- and dose-dependent manner. Neurite outgrowth appeared to be impaired at nearly all doses of chemotherapy drugs tested. iDRGOs treated with 100 nM and 50 nM bortezomib, as well as 1000 nM and 500 nM paclitaxel, exhibited no neurite outgrowth and significant neurite degeneration compared to the neurite area coverage at the start of the assay, while the same effect was observed for all concentrations of vincristine treatment (Figure 3C). While Kolliphor is an agent with known neurotoxic effects, it did not result in significant changes in neurite outgrowth at the tested doses, indicating that the effects observed with paclitaxel can be attributed to the drug.

### 3.4. Microtubule Dysregulation from Chemotherapy Drug Exposure in Sensory Neurons Differs Between Monolayer and Organoid-Based Model Systems

**Figure 4:**
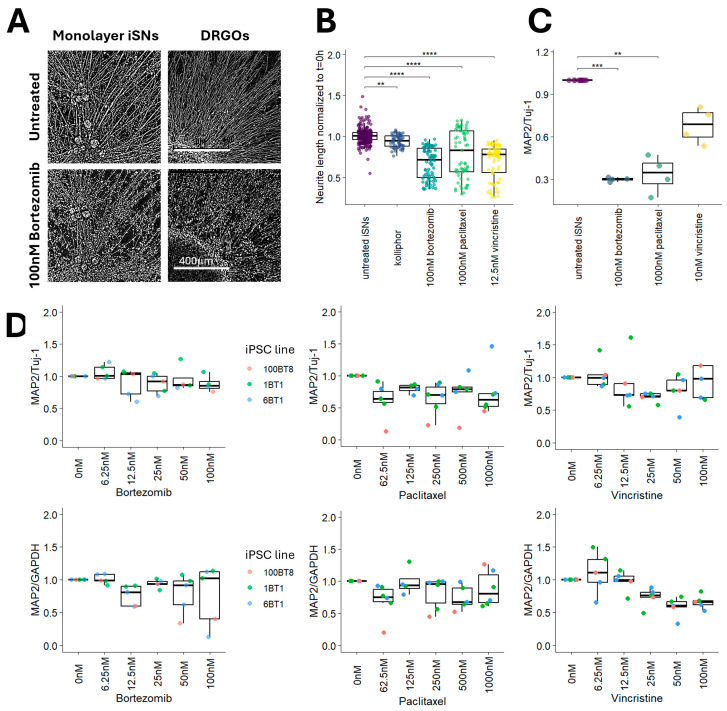
Comparison of the iDRGO model to the monolayer iSN model. (**A**) Neurite degeneration in monolayer iSNs and iDRGOs after exposure to100 nM bortezomib for 72 h. Scale bar is 400 µm. (**B**) Neurite length measurements of monolayer iSNs exposed to bortezomib, paclitaxel, vincristine, and Kolliphor (0.017%). Significance was determined using a Dunn’s test with Bonferroni correction. Four experimental batches of iSNs were assessed. (**C**) Levels of MAP2 in monolayer iSNs after 72 h of drug exposure. Significance was determined using a Dunn’s test with Holm correction. Four experimental batches of iSNs were used for each drug treatment. (**D**) Levels of MAP2 in iDRGOs exposed to chemotherapy drugs after 72 h. A Kruskal–Wallis test indicated no significant changes in MAP2 normalized to Tuj-1 between untreated iDRGOs and treated iDRGOs. Five different iDRGO batches were assessed for these protein assays. ** *p*-adj < 0.005; *** *p*-adj < 0.0005; **** *p*-adj < 0.00005.

Our previous study using monolayer iSNs to provide insights into bortezomib-induced PN indicated that a 48 h exposure leads to reduced levels of MAP2 protein, impaired axonal mitochondrial transport, and dysregulated vesicle transport from the cell soma [9]. Considering that the iSN response to chemotherapy agents may differ between monolayer and organoid models, experiments were completed to assess the levels of MAP2 as a basis for comparison. Monolayer iSNs and iDRGOs treated with 100 nM bortezomib for 72 h exhibited similar morphological characteristics of neurite stress and neurite degeneration (Figure 4A). Neurite length measurements in monolayer cultures of iSNs declined by ~30% when treated with 100 nM bortezomib, 1000 nM paclitaxel, or 12.5 nM vincristine for 72 h (Figure 4B). Although the effects of 0.017% Kolliphor were statistically significant, neurite length measurements only slightly declined (<5%) without exhibiting signs of neurite degeneration.

MAP2 protein levels were measured for iSN monolayer cultures after 72 h of chemotherapy drug exposure, and for iDRGOs after 72 h to 96 h of drug exposure. Similar to our previous findings, the MAP2/Tuj-1 ratio significantly declined with bortezomib treatment. The MAP2 levels also declined with paclitaxel or vincristine exposure (Figure 4C). For the iDRGO protein assays, the MAP2 levels were normalized to Tuj-1 as well as GAPDH to determine whether the non-neuronal cell populations could affect the response of iSNs to chemotherapy drugs within the iDRGOs. The MAP2 levels normalized to Tuj-1 did not significantly decline with drug exposure (Figure 4D); however, the MAP2/GAPDH ratio declined with 50 nM–100 nM vincristine. The Tuj-1/GAPDH ratio also declined at these concentrations (Supplemental Figure S3) which accounts for the unaffected MAP2/Tuj-1 ratio. Taken altogether, the neurotoxic effects of these chemotherapy drugs have different actions on iSNs in monolayer cultures compared to this iDRGO model.

## 4. Discussion

This study establishes a human iPSC-derived dorsal root ganglion organoid (iDRGO) platform designed for quantitative neurite outgrowth assays and evaluates whether the neurotoxic effects of common chemotherapeutics observed in two-dimensional iPSC-derived sensory neuron (iSN) cultures are recapitulated in a three-dimensional, multicellular model. The principal findings are as follows: (i) iDRGOs contain mixed neuronal and neural-crest-derived non-neuronal populations with temporal changes consistent with ongoing maturation; (ii) the iDRGO developmental stage influences the baseline neurite outgrowth capacity, emphasizing the need for assay standardization by age; (iii) bortezomib, paclitaxel, and vincristine produce drug- and dose-dependent neurite outgrowth impairment with minimal short-term effects on organoid size, consistent with early axonopathy; and (iv) microtubule-associated readouts (MAP2) diverge between monolayer and organoid contexts, suggesting that the three-dimensional architecture and cellular heterogeneity modify measurable injury phenotypes and/or drug exposure dynamics.

### 4.1. iDRGOs as a Human Sensory Dorsal-Root-Ganglion-Relevant System for CIPN Modeling

CIPN from bortezomib, paclitaxel, and vincristine is largely driven by injury to DRG sensory neurons and their axons, producing a “dying-back” pattern of distal axon degeneration that can occur with limited early neuronal loss [23,24,25]. DRGs are anatomically positioned outside the blood–brain barrier, increasing the exposure to circulating chemotherapies and contributing to selective vulnerability. In this context, iDRGOs offer a practical human system to probe early structural injury while incorporating key features absent in purified neuronal monolayers, particularly cellular heterogeneity and neuron–glia interactions. Notably, drug diffusion and penetration are more uniform in monolayer iSN cultures than in the three-dimensional iDRGO architecture, where gradients in drug exposure may arise and contribute to the observed differences in neurotoxic effects.

Using an established iDRGO differentiation protocol with modifications, iDRGOs developed a mixed cellular composition with neuronal and peripheral glial lineages, consistent with human DRG development. Markers of sensory neurons and neuronal maturity were detected alongside markers consistent with satellite glia and Schwann cell populations, and immunostaining suggested spatial heterogeneity typical of organoid systems, with neuronal markers enriched toward peripheral regions and glial markers more broadly distributed. The detailed characterization of the iDRGO model was reported by Mazzarra and colleagues [17]. Of note, the absence of overt necrosis in later-stage organoids supports the feasibility of maintaining these cultures long enough to support phenotyping and perturbation experiments.

### 4.2. Developmental Stage and Assay Window Are Major Determinants of Neurite Outgrowth

A practical insight from this work is that the organoid age influences the baseline neurite outgrowth, even when the organoid size remains stable during the assay window. Younger iDRGOs (DIV 16–22) exhibited more robust neurite outgrowth than older iDRGOs (DIV 33–38), while older organoids were larger, suggesting that time-dependent changes in cellular composition (e.g., the expansion/maturation of non-neuronal populations) and/or extracellular milieu influence the neurite outgrowth capacity. In the time-course analysis, neurite outgrowth became detectable after 24 h and continued over the subsequent days, supporting the selection of a 48 h endpoint as a balance between assay sensitivity and feasibility for scalable testing.

These observations reinforce the need to treat developmental stage as an experimental variable. For drug-testing workflows, age stratification (or restriction to a narrow DIV window) is likely to reduce variance and improve interpretability. More broadly, the developmental stage may also influence the vulnerability to chemotherapeutics via changing axon–glia signaling, trophic support, and the maturation of axonal transport and metabolic programs.

### 4.3. Chemotherapy Exposure Produces Neurite-Predominant Injury Consistent with Early Axonopathy

Across all three chemotherapies tested, neurite outgrowth was impaired in a drug- and dose-dependent manner, while the organoid size over 48 h was largely unchanged. This pattern aligns with the canonical CIPN phenotype with these drugs in which distal axons are preferentially affected early, with somatic injury and cell loss occurring later or variably depending on the agent and exposure context. Vincristine showed strong neurite suppression across the concentrations tested, while bortezomib and paclitaxel produced more graded effects with severe outgrowth failure/degeneration at higher doses. Importantly, Kolliphor did not significantly alter neurite outgrowth under these conditions, supporting the idea that the paclitaxel phenotype reflects the active drug rather than vehicle toxicity.

Functionally, these findings position the iDRGO neurite outgrowth assay as a tractable readout for early, structural CIPN-like injury, suitable for downstream mechanistic studies (e.g., axonal transport, mitochondrial dynamics, and stress pathway activation) and for prioritizing candidate neuroprotective interventions. Importantly, it is also likely that the observed reduction in neurite area reflects a combination of active neurite degeneration and impaired neurite outgrowth, rather than a single process alone.

### 4.4. Divergent MAP2 Responses Between 2D iSNs and iDRGOs Highlight Context-Dependent Phenotypes and Measurement Considerations

A key motivation for comparing monolayer iSNs and iDRGOs was to evaluate whether molecular signatures previously observed in 2D systems translate to a 3D organoid context. In monolayer iSNs, neurite degeneration after 72 h of chemotherapy exposure was accompanied by a reduced MAP2 relative to neuronal markers, consistent with prior work linking chemotherapy neurotoxicity to disruptions in cytoskeletal integrity and axonal transport [9]. In iDRGOs, despite the clear neurite impairment, the MAP2 changes were less pronounced overall, with the most evident reductions occurring with vincristine.

Several non-mutually exclusive factors may explain these model-dependent differences. First, drug penetration and exposure gradients are inherent to 3D aggregates. If neuronal somata and internal regions experience lower effective drug concentrations than neurites extending into the media, neurite degeneration may be observed without commensurate changes in bulk organoid MAP2. This differential drug exposure between neurites and somata in the 3D organoid context may lead to compartment-specific effects, such that lower effective concentrations at the soma alter the dominant pathomechanisms of neurotoxicity compared to iSNs, while also introducing distinct retrograde and anterograde signaling responses driven by spatial gradients in drug distribution. Second, cellular heterogeneity in iDRGOs necessitates normalization strategies (e.g., to GAPDH) that are appropriate for mixed populations but can dilute neuron-specific effects compared with monolayer iSNs where the neuronal purity is higher and normalization to neuronal markers is more direct. Third, neuron–glia interactions and the extracellular matrix context present in organoids may alter neuronal stress responses, cytoskeletal remodeling, and susceptibility to injury, potentially buffering or reshaping molecular endpoints even when neurite degeneration is evident. Finally, the mechanisms underlying neurotoxicity vary among the three drugs, and the aforementioned factors may further accentuate these differences between iDRGO and iSNs. This has been highlighted in a study examining bortezomib- and vincristine-induced neurotoxicity using microfluidic chambers to separate soma from neurites [21].

Rather than representing a limitation alone, these divergences emphasize an important principle: the same chemotherapeutic exposure can yield similar gross neurite phenotypes across models while engaging distinct or differently detectable molecular changes depending on the tissue context. This reinforces the value of pairing 2D and 3D human systems when prioritizing candidate mechanisms or therapeutic targets, particularly for pathways related to cytoskeletal regulation and axonal maintenance.

### 4.5. Synthesizing iDRGO Findings with Recent Human DRG and iPSC-Based CIPN Studies

Recent advances in human DRG transcriptomics and stem-cell modeling provide an important framework for interpreting iDRGO phenotypes and for positioning this platform within the current CIPN tool landscape. Lu et al. generated a spatiotemporal atlas of human embryonic DRG and leveraged these data to build functional human DRG organoids with definable sensory neuron subtypes and supporting non-neuronal lineages [26]. This work supports the concept that organoid models can approximate key features of developing human DRG and can serve as benchmarks for assessing whether iDRGOs capture the expected lineage programs and subtype composition. In parallel, LeBlang et al. demonstrated that direct contact with satellite glia promotes sensory neuron maturation and can increase susceptibility to paclitaxel-induced axonal degeneration in iPSC-derived sensory neuron systems [18]. These findings provide a compelling mechanistic rationale for why the iDRGO developmental stage and glial context may influence both baseline neurite outgrowth and chemotherapy responsiveness.

Mechanistically, convergent axon degeneration programs have been implicated across multiple neurotoxic chemotherapies, particularly involving NAD+ metabolism and Wallerian-like degeneration pathways. Geisler et al. showed that vincristine and bortezomib engage distinct upstream mechanisms but converge on a common SARM1-dependent axon degeneration program, with shared downstream features involving NMNAT2/NAD+ axis disruption [27]. Complementing this, Snavely et al. reported that bortezomib-induced neurite degeneration in human iPSC-derived neurons is associated with NAD+ depletion, is caspase-independent, and can be blocked by exogenous NAD+ in their human system [19]. These studies reinforce that early neurite degeneration in iDRGOs, particularly the neurite-predominant phenotype with a limited short-term organoid size change, fits well within a broader model in which the axonal metabolic crisis and degeneration pathways are engaged early, even when the soma viability appears preserved.

Finally, the prior iPSC-based discovery work highlights the value of scalable neurite phenotyping for screening and biomarker identification. Hrstka et al. used proteomics in human iSNs to implicate cell stress responses and microtubule dynamics dysfunction in bortezomib neurotoxicity, with prominent effects on MAP2 isoforms and proteins involved in transport and homeostasis [9]. Petrova et al. developed a high-content screening paradigm for vincristine-induced neurotoxicity in iPSC-derived neurons and identified small molecules with neuroprotective activity [28]. Together with the mechanistic work above, these studies suggest that iDRGO neurite-outgrowth assays, paired with an automated image analysis, could be adapted for the medium-throughput prioritization of candidate neuroprotectants and for the hypothesis-driven testing of pathways such as NAD+ metabolism, axonal transport, and stress signaling in a more tissue-like 3D context.

### 4.6. Limitations and Future Directions

This study should be interpreted in light of several important limitations that highlight the need for more detailed mechanistic investigations. First, although a major advantage of the iDRGO system is the inclusion of non-neuronal cell populations, the current study does not directly define how chemotherapeutic agents differentially affect neuronal versus non-neuronal cells or how their interactions shape the overall neurotoxicity. Given that glial- and other neural-crest-derived cells can influence neuronal maturation, stress responses, and injury susceptibility, future work should incorporate cell-type-specific analyses, including viability, mitochondrial function, oxidative stress, and signaling pathways, to better define their contributions to CIPN. Second, iDRGOs remain developmentally immature relative to adult human DRG, and their cellular composition evolves over time. The organoids used for functional assays (DIV 16–37) were not characterized at the same depth as later-stage organoids, introducing uncertainty regarding the specific cell populations contributing to observed phenotypes. Time-matched, high-resolution profiling (e.g., single-cell or spatial transcriptomics) will be important to address this limitation. Third, protein quantification from heterogeneous organoid lysates may obscure cell-type-specific or compartment-specific changes, particularly when early injury is localized to neurites. Normalization approaches suitable for mixed populations may dilute neuron-specific signals compared to monolayer systems. Fourth, our iDRGO in vitro system does not distinguish between drug effects on neuronal soma versus neurites. In vivo, these compartments experience different exposure environments, whereas, in iDRGOs, both are directly exposed, albeit at different concentrations. This may alter the dominant mechanisms of neurotoxicity and the balance of retrograde and anterograde injury signaling. Compartmentalized systems, such as microfluidic platforms, will be important for resolving these differences. Fifth, the study focuses on early structural phenotypes (48–96 h), whereas clinical CIPN reflects cumulative exposure. In addition, organoid size is a limited surrogate for cell viability, and more direct assessments will be needed to define somatic and non-neuronal injury. Finally, although normalization strategies were used, variability in the developmental stage remains a potential confounder.

Taken together, these limitations highlight that the current work serves as a foundational platform rather than a fully resolved mechanistic model. Future studies should prioritize (i) cell-type-specific and spatially resolved analyses of drug response; (ii) a direct comparison of neuronal and non-neuronal cell vulnerability; (iii) compartmentalized exposure paradigms to model soma versus axon toxicity; and (iv) the incorporation of functional and molecular readouts over longer exposure periods. Such approaches will be critical for defining how chemotherapy-induced injury emerges from the interplay between neuronal and non-neuronal cells and for improving the translational relevance of human iPSC-derived DRG models in CIPN research.

## Data Availability

The original contributions presented in this study are included in the article/Supplementary Material. Further inquiries can be directed to the corresponding authors.

## Supplementary Materials

The following supporting information can be downloaded at: https://www.mdpi.com/article/10.3390/cells15080724/s1; Figure S1: Immunofluorescent characterization of neuronal and glial markers; Figure S2: Neurite outgrowth in DMSO; Figure S3: Tuj-1 levels in treated iDRGO; Figure S4: Protein assays images from the characterization of iDRGOs in Figure 1C.; Figure S5: Protein assay images from the chemotherapy drug experiments with monolayer iSNs in Figure 4C; Figure S6: Protein assay images from the chemotherapy drug experiments in Figure 4D. Table S1: Primers for q-PCR experiments; Table S2: List of reagents used for cell culture, antibodies, and experiments; Table S3: List of images used for iDRGO characterization; Table S4: List of images used for the chemotherapy drug experiments with monolayer iSNs; Table S5: List of images used for the chemotherapy drug experiments with iDRGOs.
