# Supplementary material for: Human iPSC-Derived Dorsal Root Ganglion Organoid Modeling of Chemotherapy-Induced Peripheral Neuropathy
Source: Cells. 2026 Apr 19;15(8):724. doi: 10.3390/cells15080724 (PMC13115049; doi:10.3390/cells15080724)
Supplement: Supplementary file 1 [file cells-15-00724-s001.zip › cells-4229431-supplementary.pdf]

Supplementary Materials for "Human iPSC-derived dorsal root ganglion organoid modelling of chemotherapy-induced peripheral neuropathy" by Hrstka et al.

Supplementary Table S1. Primers for q-PCR experiments.

**Gene**	**Left**	**Right**	**Product Size**	**Temp**	**Location of Primer**
**SOX10**	GAGTTGGACCAGTACCTGCC	GTCTCTGTCTTCACCTGGGC	194	60.04. 60.04	
**FABP7**	GGCTTTCTGTGCTACCTGGA	TCAAACTCTTCTCCCAGCTGG	399	59.68, 59.65	
**GFAP**	GAAGCAGATGAAGCCACCCT	CATTGCCTCATACTGCGTGC	213	60.03, 59.97	
**S100B**	CCTCATCGACGTTTTCCACC	GAAGTCACATTCGCCGTCTC	279	58.92, 59.01	
**TUBB3**	GCCTCAAGATGTCCTCCACC	CGTACATCTCGCCCTCTTCC	234	60.11, 59.97	
**MAP2 Transcript Variant 5**	CTATCCCAGGACCCCTCACA	CTTCAGGTCTGGCAGTGGTT	151	60.03, 59.89	966-986, 1096-1116
**NMNAT2**	TCTTGGGGAAGGTGGGAGAA	TCACTACCACACAGCAGCAG	151	60.10, 59.97	440-460, 570-590

Supplementary Table S2. List of reagents used for cell culture, antibodies, and experiments.

Reagent	Catalog #	Application & Usage	Source
MAP2 Polyclonal Antibody	17490-1-AP	IF (1:500); WB (1:125)	Proteintech
Tuj-1 (neuron-specific class III beta-tubuin) Monoclonal Antibody	66375-1-Ig	IF (1:1000)	Proteintech
TUBB3 Antibody		WB (1:1000)	Biolegend
UCHL1/PGP9.5 Polyclonal Antibody	14730-1-AP	IF (1:200); WB (1:1000)	Proteintech
FABP7 Polyclonal Antibody	PA5-24949	IF (1:200); WB (1:1000)	Invitrogen
GFAP Monoclonal Antibody	60190-1-Ig	IF (1:500); WB (1:2000)	Proteintech
GAPDH Polyclonal Antibody		WB (1:10)	Proteintech
Nano Secondary alpaca anti-rabbit IgG recombinant VHH, Alexa Fluor 647	srb2GCL647	IF (1:500)	Proteintech
Nano Secondary alpaca anti-mouse IgG1 recombinant VHH, Alexa Fluor 488	sms1AF488	IF (1:500)	Proteintech
Nano-Secondary anti-mouse IgG1, recombinant VHH, CoraLite® Plus 647	smsG1CL647	IF (1:500)	Proteintech
**Cell Culture & Biochemistry Reagents**			
Bortezomib (PS-341), Free Base, >99%	B-1408	6.25nM - 100 nM	LC laboratories
Vincristine	S1241	6.25nM - 100 nM	Selleck Chemicals
DMSO	D2650	≤ 0.0001% (bortezomib)<br>≤ 0.002% (vincristine)	Sigma-Aldrich
mTeSR™ 1	85850		STEMCELL Technologies Inc.
KnockOut™ DMEM	10829018		Life Technologies
KnockOut™ Serum Replacement (15%)	10828028	15%	Life Technologies
GlutaMAX™ Supplement (100X)	35050061	1%	Life Technologies
MEM Non-Essential Amino Acids Solution (100X)	11140050	1%	Life Technologies
2-Mercaptoethanol	21985023	55 Mm, 100uM	Life Technologies
Accutase	AT-104		Innovative Cell Technologies
Y-27632	5092280001	10 ng/ml	Calbiochem
LDN193189	SML0559	100 nM	Sigma-Aldrich
SB431542	Ab120163	10 µM	abcam
CHIR99021	4423	3 µM	Tocris Bioscience
DAPT	2634	10 µM	Tocris Bioscience
SU5402	SML0443	5 µM	Sigma-Aldrich
Recombinant human β-NGF	256-GF-100	25 ng/ml	R&D Systems
Recombinant BDNF	248-BD-005	25 ng/ml	R&D Systems
Recombinant human GDNF	212-GD-010	25 ng/ml	R&D Systems
Recombinant NT-3	267-N3-025	25 ng/ml	R&D Systems
cAMP	D0627	0.5 mM	Sigma-Aldrich
L-ascorbic acid	A8960	200 µM	Sigma-Aldrich
DAPI	D1306		Invitrogen

16% Paraformaldehyde (formaldehyde) aqueous solution	15710	4%	Electron Microscopy Sciences
Triton™ X-100	X100-100ML	0.5%	Sigma-Aldrich
SuperBlock™ (PBS) Blocking Buffer	37580	+ 0.05% Triton X-100	Thermo Scientific™
ProLong antifade mountant	P36930		Invitrogen
Halt™ Protease Inhibitor Cocktail (100X)	87786		Thermo Scientific™
Halt™ Protease and Phosphatase Inhibitor Cocktail (100X)	78446		Thermo Scientific™
Benzonase® Nuclease	E1404		MilliporeSigma
Geltrex™ LDEV-Free, hESC-Qualified, Reduced Growth Factor Basement Membrane Matrix	A1413302		Gibco®
12-230 kDa Jess or Wes Separation Module	SM-W004		proteinsimple
66-440 kDa Jess or Wes Separation Module	SM-W008		proteinsimple
Anti-Rabbit Detection Module	DM-001		proteinsimple
Anti-Mouse Detection Module	DM-002		proteinsimple

Supplementary Table S3. Key for protein assays: characterization of selected markers.

**iDRGO characterization westerns**			
**marker**	**blot**	**lane(s)**	**file name**
TUJ1	Blot 1	15	Blot 1 Fig1C_full_blot_TUJ1
MAP2|FABP7|NMNAT2	Blot 2	9, 12	Blot 2 Fig_1C_full_blot_MAP2_FABP7_NMNAT2_labeled
PGP9.5	Blot 3	18	Blot 3 Fig_1C_full_blot_PGP9.5
GFAP	Blot 4	20	Blot 4 Fig_1C_full_blot2_GFAP_labeled
S100B	Blot 5	7	Blot 5 Fig_1C_full_blot3_S100B_labeled

Supplemental Table S4. Key for protein assays: iSN monolayer experiments with chemotherapy drugs.

**iSN westerns**			
**iPSC line**	**drug**	**blot**	**file name**
**1BT1**	**bortezomib**	**Blot 10**	**Bortezomib_all_3_lines_blot_10**
**6BT1**	**bortezomib**	**Blot 10**	**Bortezomib_all_3_lines_blot_10**
**100BT8 batch 2**	**bortezomib**	**Blot 10**	**Bortezomib_all_3_lines_blot_10**
**100BT8 batch 1**	**bortezomib**	**Blot 7**	**100BT8_bortezomib_blot_7**
**6BT1 batch 1**	**vincristine**	**Blot 12**	**6BT1_1BT1_vincristine_blot_12**
**6BT1 batch 2**	**vincristine**	**Blot 12**	**6BT1_1BT1_vincristine_blot_12**
**1BT1**	**vincristine**	**Blot 12**	**6BT1_1BT1_vincristine_blot_12**
**100BT8**	**vincristine**	**Blot 8**	**100BT8_vincristine_blot_8**
**100BT8 batch 1**	**paclitaxel**	**Blot 9**	**6BT1_100BT8_Paclitaxel_blot_9**
**100BT8 batch 2**	**paclitaxel**	**Blot 11**	**100BT8_paclitaxel_blot_11**
**6BT1**	**paclitaxel**	**Blot 9**	**6BT1_100BT8_Paclitaxel_blot_9**
**1BT1**	**paclitaxel**	**Blot 6**	**1BT1_paclitaxel_blot_6**

Supplemental Table S5. Key for protein assays: iDRGO experiments with chemotherapy drugs.

**iDRGO westerns**			
**iPSC line**	**drug**	**blot**	**file name**
1BT1 batch 1	bortezomib	Blot 13	1BT1_6BT1_bortezomib_iDRGO_blot_13
1BT1 batch 2	bortezomib	Blot 14	1BT1_6BT1_iDRGO_bortezomib_vincristine_blot_14
6BT1 batch 1	bortezomib	Blot 13	1BT1_6BT1_bortezomib_iDRGO_blot_13
6BT1 batch 2	bortezomib	Blot 14	1BT1_6BT1_iDRGO_bortezomib_vincristine_blot_14
100BT8	bortezomib	Blot 18	1BT1_6BT1_100BT8_iDRGO_bortezomib_paclitaxel_kolliphor_blot_18
1BT1 batch 1	vincristine	Blot 14	1BT1_6BT1_iDRGO_bortezomib_vincristine_blot_14
1BT1 batch 2	vincristine	Blot 15	1BT1_6BT1_iDRGO_vincristine_paclitaxel_blot_15
6BT1 batch 1	vincristine	Blot 14	1BT1_6BT1_iDRGO_bortezomib_vincristine_blot_14
6BT1 batch 2	vincristine	Blot 15	1BT1_6BT1_iDRGO_vincristine_paclitaxel_blot_15
100BT8	vincristine	Blot 17	1BT1_6BT1_100BT8_iDRGO_paclitaxel_vincristine_kolliphor_blot_17
1BT1 batch 1	paclitaxel	Blot 15	1BT1_6BT1_iDRGO_vincristine_paclitaxel_blot_15
1BT1 batch 2	paclitaxel	Blot 15	1BT1_6BT1_iDRGO_vincristine_paclitaxel_blot_15
6BT1 batch 1	paclitaxel	Blot 16	1BT1_6BT1_iDRGO_paclitaxel_kolliphor_blot_16
6BT1 batch 2	paclitaxel	Blot 17	1BT1_6BT1_100BT8_iDRGO_paclitaxel_vincristine_kolliphor_blot_17
100BT8	paclitaxel	Blot 17	1BT1_6BT1_100BT8_iDRGO_paclitaxel_vincristine_kolliphor_blot_17
1BT1 batch 3	paclitaxel	Blot 18	1BT1_6BT1_100BT8_iDRGO_bortezomib_paclitaxel_kolliphor_blot_18
1BT1 batch 1	kolliphor	Blot 16	1BT1_6BT1_iDRGO_paclitaxel_kolliphor_blot_16
1BT1 batch 2	kolliphor	Blot 17	1BT1_6BT1_100BT8_iDRGO_paclitaxel_vincristine_kolliphor_blot_17
6BT1 batch 1	kolliphor	Blot 16	1BT1_6BT1_iDRGO_paclitaxel_kolliphor_blot_16
6BT1 batch 2	kolliphor	Blot 18	1BT1_6BT1_100BT8_iDRGO_bortezomib_paclitaxel_kolliphor_blot_18
100BT8	kolliphor	Blot 18	1BT1_6BT1_100BT8_iDRGO_bortezomib_paclitaxel_kolliphor_blot_18

Supplmentary Figure S1

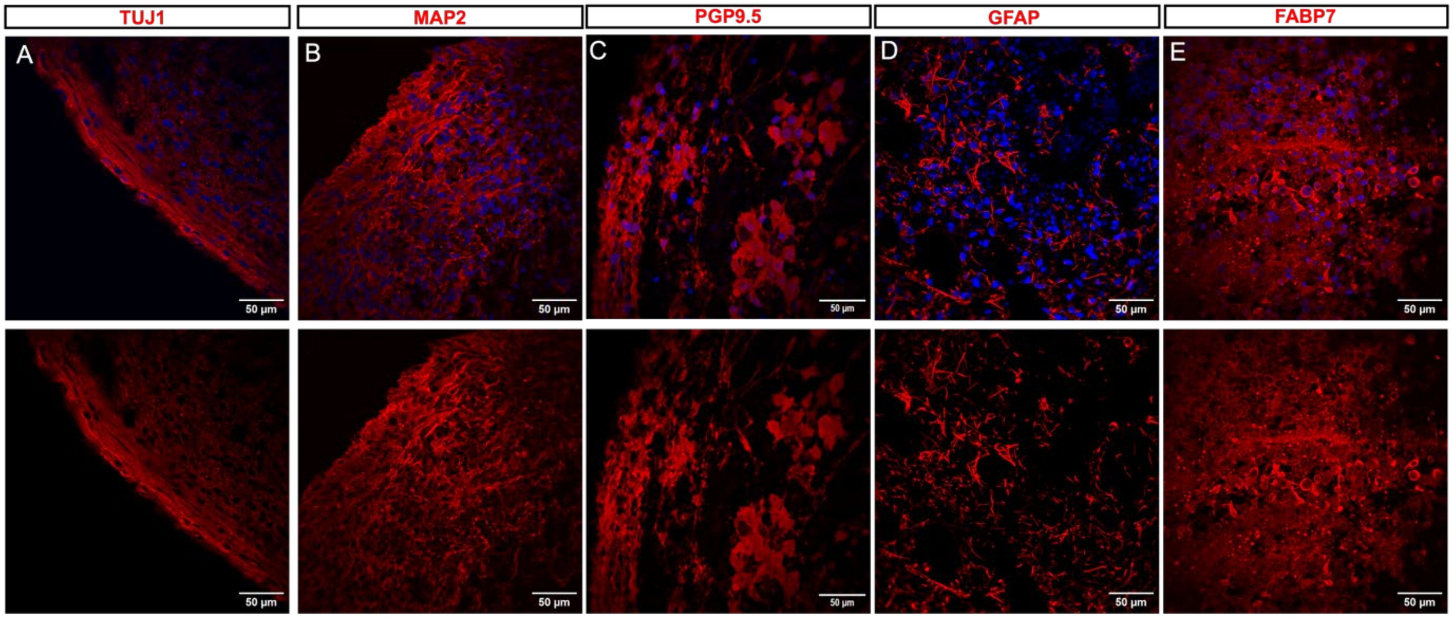

Supplemental Figure S1. Immunofluorescent Characterization of Neuronal and Glial Cell Markers. Representative immunofluorescence images of 10m iDRGO sections stained for (A) TUJ1, (B) MAP2, (C) PGP9.5, (D) GFAP, (E) FABP7. Images were taken with a 4x water immersion objective.

Supplementary Figure S2

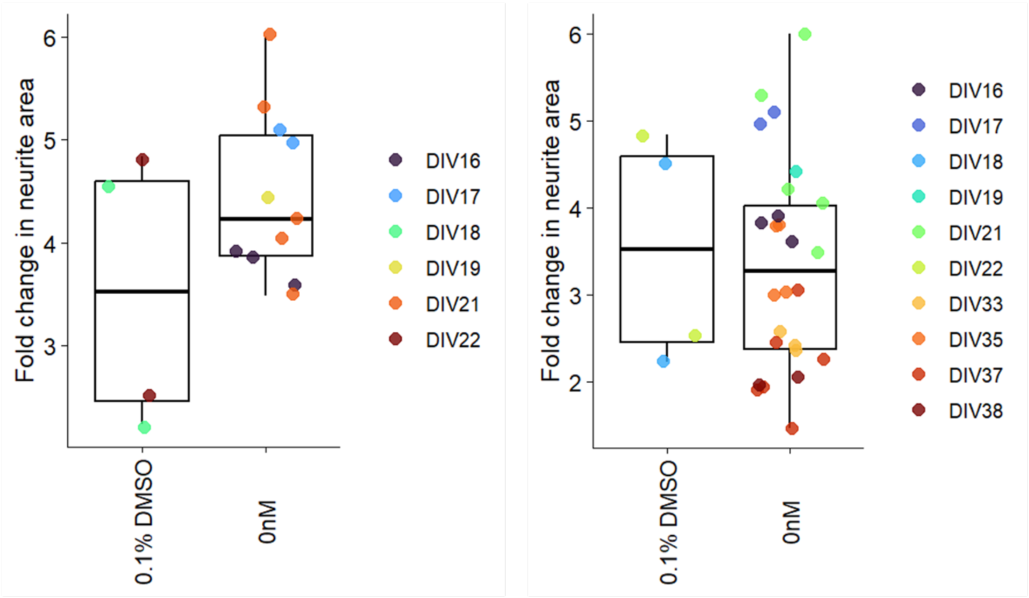

Supplemental figure S2. Neurite outgrowth in DIV18 – DIV22 iDRGOs is unaffected by 0.1% DMSO after 48h (n = 4). A Kruskal-Wallis test indicated no significant difference between treated and untreated iDRGOs.

Supplementary Figure S3.

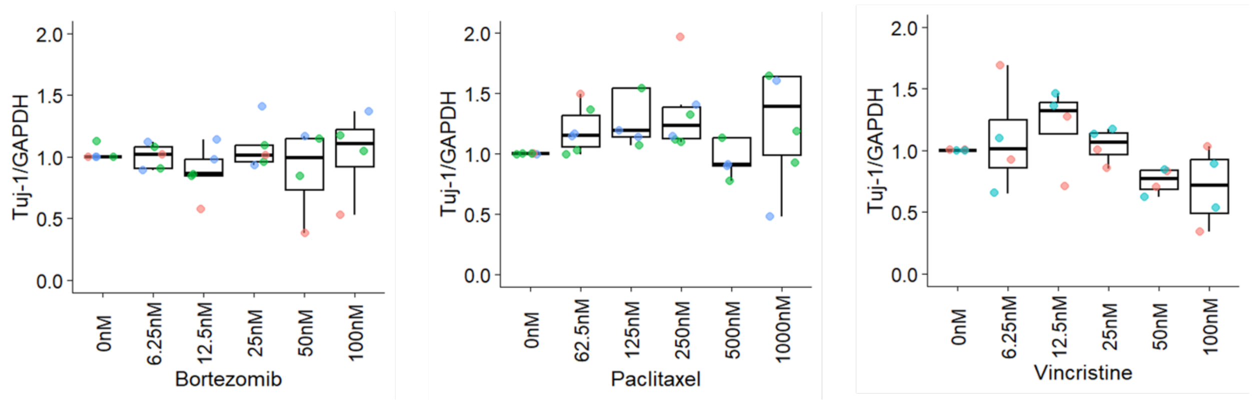

Supplemental figure S3. Levels of Tuj-1/GAPDH in iDRGOs treated with bortezomib, paclitaxel, or vincristine for 72h – 96h. Kruskal-Wallis test indicated no significant difference between treated and untreated iDRGOs. 5 experimental baches were assessed in these protein assays.

Supplemental Figure S4. Protein assays from the characterization of iDRGOs in Figure 1C.

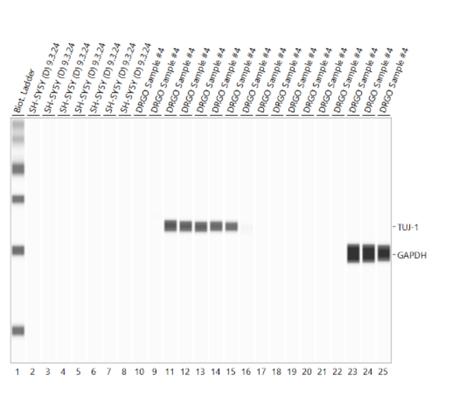

Blot 1: Fig1C_full_blot_TUJ1

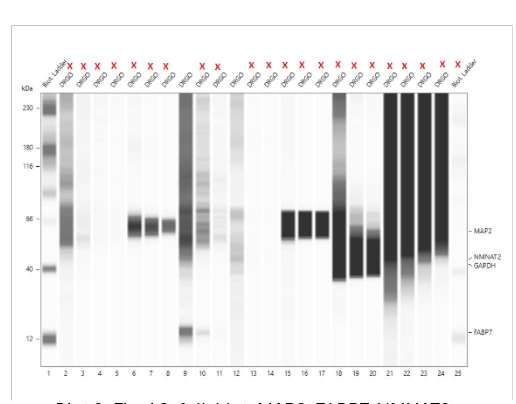

Blot 2: Fig_1C_full_blot_MAP2_FABP7-NMNAT2

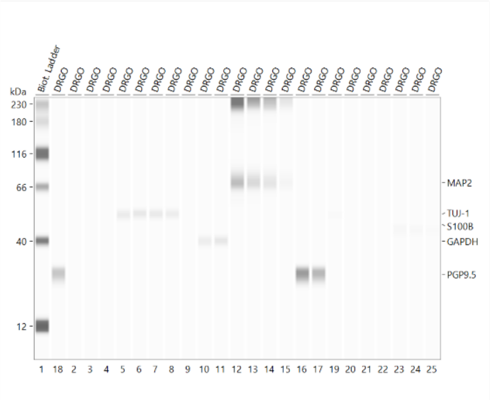

Blot 3: Fig_1C_full_blot_PGP9.5

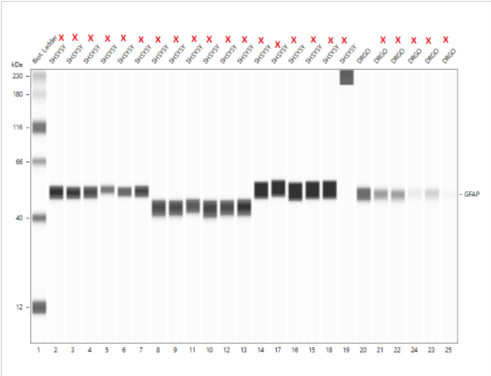

Blot 4: Fig_1C_full_blot2_GFAP_labeled

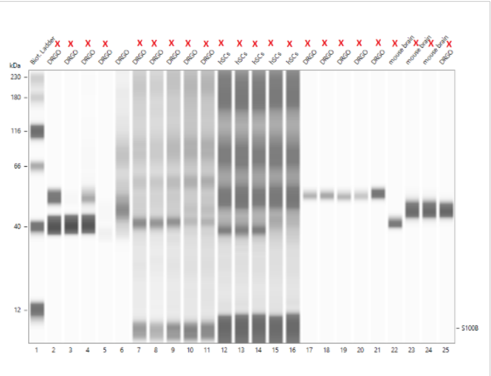

Blot 5: Fig_1C_full_blot3_S100B_labeled

Supplemental figure S5. Protein assays from the chemotherapy drug experiments with iSNs from Figure 4C.

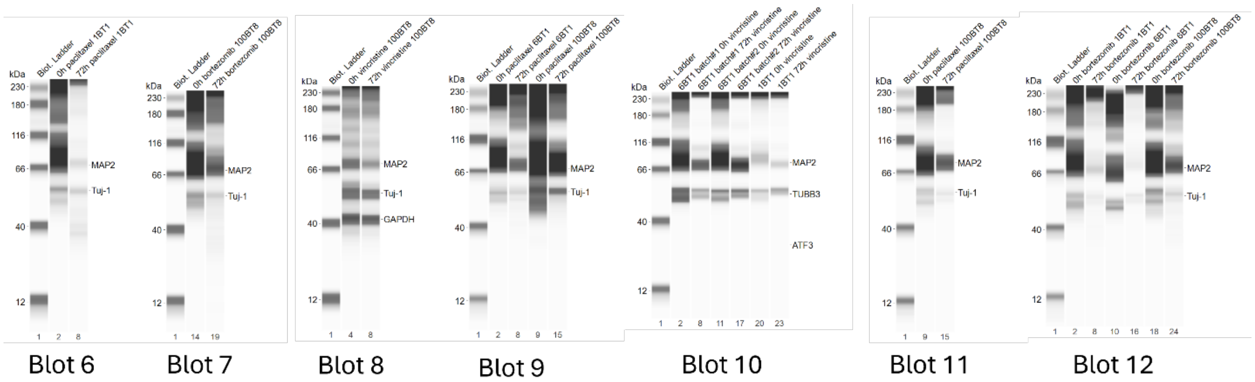

Supplemental figure S6. Protein assays from the chemotherapy drug experiments with iDRGOs from Figure 4D.

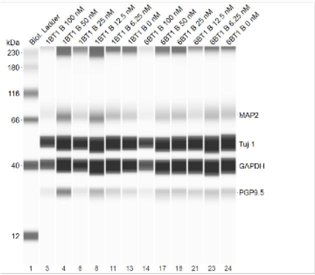

Blot 13

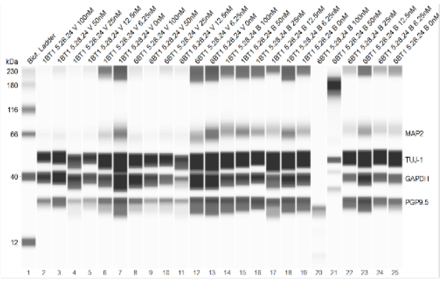

Blot 14

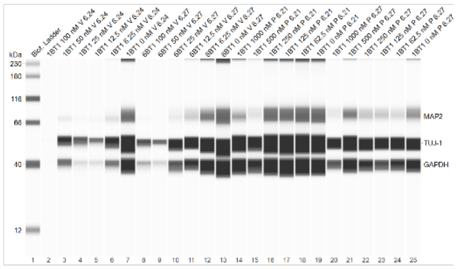

Blot 15

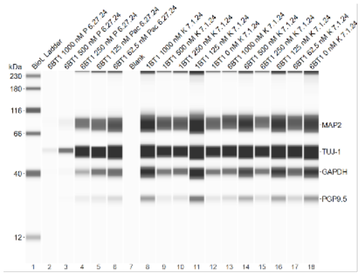

Blot 16

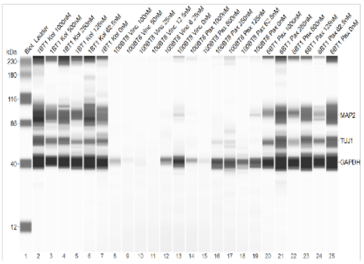

Blot 17

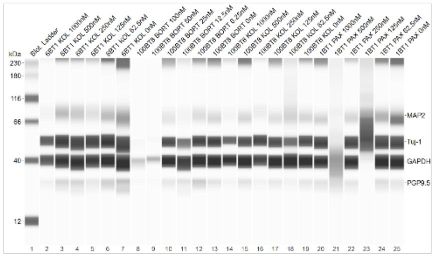

Blot 18
